# Supplementary material for: Syntrophic Hydrocarbon Degradation in a Decommissioned Off-Shore Subsea Oil Storage Structure
Source: Microorganisms. 2021 Feb 11;9(2):356. doi: 10.3390/microorganisms9020356 (PMC7916938; doi:10.3390/microorganisms9020356)
Supplement: Supplementary file 1 [file microorganisms-09-00356-s001.zip › Supplementary Table S1.docx]

| Target | | Method | Technique | Units | Limit of Detection | Cell6-3 Results | Cell6-4 Results |
| --- | --- | --- | --- | --- | --- | --- | --- |
| Total Petroleum Hydrocarbon C6 | | TNRCC1005 | GC/FID | mg/L | 0.5 | 2.2 | 1.8 |
| Total Petroleum Hydrocarbon >C6-C8 | | TNRCC1005 | GC/FID | mg/L | 0.5 | 4.7 | 3.8 |
| Total Petroleum Hydrocarbon >C8-C10 | | TNRCC1005 | GC/FID | mg/L | 0.5 | 1.3 | 1.1 |
| Total Petroleum Hydrocarbon >C10-C12 | | TNRCC1005 | GC/FID | mg/L | 0.5 | 0.9 | 0.8 |
| Total Petroleum Hydrocarbon >C12-C16 | | TNRCC1005 | GC/FID | mg/L | 0.5 | 1.8 | 1.7 |
| Total Petroleum Hydrocarbon >C16-C21 | | TNRCC1005 | GC/FID | mg/L | 0.5 | 2.7 | 2.0 |
| Total Petroleum Hydrocarbon >C21-C35 | | TNRCC1005 | GC/FID | mg/L | 0.5 | 9.0 | 7.1 |
| Total Petroleum Hydrocarbon >C35->C40 | | TNRCC1005 | GC/FID | mg/L | 0.5 | 1.6 | 1.2 |
| Total Petroleum Hydrocarbon C>40 | | TNRCC1005 | GC/FID | mg/L | 0.5 | <0.5 | <0.5 |
| Total Petroleum Hydrocarbon C5-C>40 | | TNRCC1005 | GC/FID | mg/L | 0.5 | 24.3 | 19.4 |
| Total Petroleum Hydrocarbon C>5-C6 Aliphatic | | 8260C | GC/MS/HS | mg/L | 0.5 | 0.9 | 0.9 |
| Total Petroleum Hydrocarbon C>6-C8 Aliphatic | | TNRCC1006 | GC/FID | mg/L | 0.5 | 2.1 | 1.2 |
| Total Petroleum Hydrocarbon C>8-C10 Aliphatic | | TNRCC1006 | GC/FID | mg/L | 0.5 | 0.6 | <0.5 |
| Total Petroleum Hydrocarbon C>10-C12 Aliphatic | | TNRCC1006 | GC/FID | mg/L | 0.5 | <0.5 | <0.5 |
| Total Petroleum Hydrocarbon C>12-C16 Aliphatic | | TNRCC1006 | GC/FID | mg/L | 0.5 | 0.9 | 0.9 |
| Total Petroleum Hydrocarbon C>16-C21 Aliphatic | | TNRCC1006 | GC/FID | mg/L | 0.5 | 1.1 | 1.1 |
| Total Petroleum Hydrocarbon C>21-C35 Aliphatic | | TNRCC1006 | GC/FID | mg/L | 0.5 | 2.1 | 1.5 |
| Total Petroleum Hydrocarbon C>35-C40 Aliphatic | | TNRCC1006 | GC/FID | mg/L | 0.5 | <0.5 | <0.5 |
| Total Petroleum Hydrocarbon C>40 Aliphatic | | TNRCC1006 | GC/FID | mg/L | 0.5 | <0.5 | <0.5 |
| Total Petroleum Hydrocarbon C>5-C>40 Aliphatic | | TNRCC1006 | GC/FID | mg/L | 4.00 | 8.2 | 6.5 |
| Total Petroleum Hydrocarbon C>6-C8 Aromatic | | TNRCC1006 | GC/FID | mg/L | 0.5 | 0.7 | 1.7 |
| Total Petroleum Hydrocarbon C>8-C10 Aromatic | | TNRCC1006 | GC/FID | mg/L | 0.5 | <0.5 | 0.5 |
| Total Petroleum Hydrocarbon C>10-C12 Aromatic | | TNRCC1006 | GC/FID | mg/L | 0.5 | <0.5 | <0.5 |
| Total Petroleum Hydrocarbon C>12-C16 Aromatic | | TNRCC1006 | GC/FID | mg/L | 0.5 | <0.5 | <0.5 |
| Total Petroleum Hydrocarbon C>16-C21 Aromatic | | TNRCC1006 | GC/FID | mg/L | 0.5 | <0.5 | <0.5 |
| Total Petroleum Hydrocarbon C>21-C35 Aromatic | | TNRCC1006 | GC/FID | mg/L | 0.5 | 0.7 | 0.6 |
| Total Petroleum Hydrocarbon C>35-C40 Aromatic | | TNRCC1006 | GC/FID | mg/L | 0.5 | <0.5 | <0.5 |
| Total Petroleum Hydrocarbon C>40 Aromatic | | TNRCC1006 | GC/FID | mg/L | 0.5 | <0.5 | <0.5 |
| Total Petroleum Hydrocarbon C>5-C>40 Aromatic | | TNRCC1006 | GC/FID | mg/L | 4.00 | <4.0 | <4.0 |
| Total Petroleum Hydrocarbon C>5-C>40 Ali+Aro | | TNRCC1006 | GC/FID | mg/L | 8.00 | 11.1 | 10.2 |
| Naphthalene | | US EPA 625 | GC/MS | µg/L | 100 | **132** | **123** |
| Acenaphthylene | | US EPA 625 | GC/MS | µg/L | 100 | <100 | <100 |
| Acenaphthene | | US EPA 625 | GC/MS | µg/L | 100 | <100 | <100 |
| Fluorene | | US EPA 625 | GC/MS | µg/L | 100 | <100 | <100 |
| Phenanthrene | | US EPA 625 | GC/MS | µg/L | 100 | <100 | <100 |
| Anthracene | | US EPA 625 | GC/MS | µg/L | 100 | <100 | <100 |
| Fluoranthene | | US EPA 625 | GC/MS | µg/L | 100 | <100 | <100 |
| Pyrene | | US EPA 625 | GC/MS | µg/L | 100 | <100 | <100 |
| Benz(a)anthracene | | US EPA 625 | GC/MS | µg/L | 100 | <100 | <100 |
| Chrysene | | US EPA 625 | GC/MS | µg/L | 100 | <100 | <100 |
| Benzo(b/j)fluoranthene | | US EPA 625 | GC/MS | µg/L | 100 | <100 | <100 |
| Benzo(k)fluoranthene | | US EPA 625 | GC/MS | µg/L | 100 | <100 | <100 |
| Benzo(a)pyrene | | US EPA 625 | GC/MS | µg/L | 100 | <100 | <100 |
| Indeno(1,2,3-CD)pyrene | | US EPA 625 | GC/MS | µg/L | 100 | <100 | <100 |
| Dibenz(ah)anthracene | | US EPA 625 | GC/MS | µg/L | 100 | <100 | <100 |
| Benzo(ghi)perylene | | US EPA 625 | GC/MS | µg/L | 100 | <100 | <100 |
| Benzo(a)fluoranthene | | US EPA 625 | GC/MS | µg/L | 100 | <100 | <100 |
| Benzo(e)pyrene | | US EPA 625 | GC/MS | µg/L | 100 | <100 | <100 |
| Perylene | | US EPA 625 | GC/MS | µg/L | 100 | <100 | <100 |
| Dibenzo(a,l)pyrene | | US EPA 625 | GC/MS | µg/L | 100 | <100 | <100 |
| Dibenzo(a,i)pyrene | | US EPA 625 | GC/MS | µg/L | 100 | <100 | <100 |
| Dibenzo(a,h)Pyrene | | US EPA 625 | GC/MS | µg/L | 100 | <100 | <100 |
| Dibenzo(a,e)pyrene | | US EPA 625 | GC/MS | µg/L | 100 | <100 | <100 |
| Beryllium | Be | US EPA 200.7 | ICP-MS | µg/L | 0.05 | <0.05 | <0.05 |
| Magnesium | Mg | US EPA 200.7 | ICP-MS | µg/L | 20 | 560 | 563 |
| Aluminium | Al | US EPA 200.7 | ICP-MS | µg/L | 1.5 | <1.5 | <1.5 |
| Vanadium | V | US EPA 200.7 | ICP-MS | µg/L | 0.09 | <0.09 | <0.09 |
| Chromium | Cr | US EPA 200.7 | ICP-MS | µg/L | 0.12 | <0.12 | <0.12 |
| Manganese | Mn | US EPA 200.7 | ICP-MS | µg/L | 0.41 | <0.41 | <0.41 |
| Iron | Fe | US EPA 200.7 | ICP-MS | µg/L | 6.7 | <6.7 | <6.7 |
| Cobalt | Co | US EPA 200.7 | ICP-MS | µg/L | 0.03 | <0.03 | <0.03 |
| Nickel | Ni | US EPA 200.7 | ICP-MS | µg/L | 1.4 | <1.4 | <1.4 |
| Copper | Cu | US EPA 200.7 | ICP-MS | µg/L | 0.16 | <0.16 | <0.16 |
| Zinc | Zn | US EPA 200.7 | ICP-MS | µg/L | 12 | <12 | <12 |
| Arsenic | As | US EPA 200.7 | ICP-MS | µg/L | 0.09 | <0.09 | <0.09 |
| Selenium | Se | US EPA 200.7 | ICP-MS | µg/L | 0.14 | <0.14 | <0.14 |
| Strontium | Sr | US EPA 200.7 | ICP-MS | µg/L | 1.6 | 28.97 | 28.71 |
| Molybdenum | Mo | US EPA 200.7 | ICP-MS | µg/L | 0.18 | <0.18 | <0.18 |
| Cadmium | Cd | US EPA 200.7 | ICP-MS | µg/L | 0.04 | <0.04 | <0.04 |
| Antimony | Sb | US EPA 200.7 | ICP-MS | µg/L | 0.05 | <0.05 | <0.05 |
| Barium | Ba | US EPA 200.7 | ICP-MS | µg/L | 1.1 | 4.16 | 4.92 |
| Thallium | Tl | US EPA 200.7 | ICP-MS | µg/L | 0.85 | <0.85 | <0.85 |
| Lead | Pb | US EPA 200.7 | ICP-MS | µg/L | 0.09 | <0.09 | <0.09 |
| Thorium | Th | US EPA 200.7 | ICP-MS | µg/L | 0.79 | <0.79 | <0.79 |
| Mercury | Hg | BS EN ISO 17852 | Atomic Fluorescence | ng/L | 0.01 | 980 | 980 |
| pH | | BS ISO 10523 | Probe | pH | - | 7.9 | 7.9 |
| Temperature at time of pH measurement | | BS ISO 10523 | Probe | ^o^C | - | 20.1 | 21.1 |
| Electrical Conductivity ϒ_25_ | | EN 27888 | Probe | µS/cm | - | 45500 | 45600 |
| Temperature at time of conductivity measurement | | EN 27888 | Probe | ^o^C | - | 21.1 | 21.3 |
| Redox Potential | | ASTM D1498 | Probe | mV | - | -31.8 | -31.7 |
| Sample Temp at time of redox measurement | | ASTM D1498 | Probe | ^o^C | - | 20.1 | 21.1 |
| pH at time of redox measurement |  | ASTM D1498 | Probe | pH | - | 7.9 | 7.9 |
| Density at 15^o^C | | ASTM D4052 | Digital Densitometer | g/ml | - | 1.0219 | 1.0219 |
| Total Alkalinity ** | | EN ISO 9963 | Titration | mmol(H+)/L | - | 29 | 30 |
| Total Suspended Solids (glass microfibre) ** | | BS EN 872 | Filtration | mg/L | 40 | 220 | 200 |
| Benzene | | US EPA 624 | GC/MS | µg/L | 1 | 3260 | 3180 |
| Toluene | | US EPA 624 | GC/MS | µg/L | 1 | 1240 | 1180 |
| Ethyl Benzene | | US EPA 624 | GC/MS | µg/L | 1 | 63 | 60 |
| m/p Xylene | | US EPA 624 | GC/MS | µg/L | 1 | 250 | 230 |
| o Xylene | | US EPA 624 | GC/MS | µg/L | 1 | 160 | 160 |
| Most Probable Number SRB | | AM-P-097A | - | cells/ml | 0.3 | <0.3 | <0.3 |
| Most Probable Number SRB 21 days | | AM-P-097A | - | cells/ml | 0.3 | <0.3 | <0.3 |
| Most Probable Number NRB | | AM-P-097C | - | cells/ml | 0.3 | <0.3 | <0.3 |
| Hetrotropic Bacteria Count @ 37^o^C after 44 hours | | AM-P-DWI-007 | - | cfu/ml | 1 | <1 | <1 |
| Hetrotropic Bacteria Count @ 22^o^C after 68 hours | | AM-P-DWI-007 | - | cfu/ml | 1 | <1 | <1 |
| Fluoride | | ISO 10304 | IC | mg/L | 2 | <2.0 | <2.0 |
| Chloride | | ISO 10304 | IC | mg/L | 2 | 18750 | 18750 |
| Nitrate | | ISO 10304 | IC | mg/L | 2 | <2.0 | <2.0 |
| Nitrite | | ISO 10304 | IC | mg/L | 2 | <2.0 | <2.0 |
| Phosphate | | ISO 10304 | IC | mg/L | 2 | <2.0 | <2.0 |
| Bromide | | ISO 10304 | IC | mg/L | 2 | 56 | 55 |
| Sulphate | | ISO 10304 | IC | mg/L | 2 | 1010 | 1000 |
| Acetic Acid | | US EPA 624 mod | GC/MS | mg/L | 0.1 | <0.1 | <0.1 |
| Propanoic Acid | | US EPA 624 mod | GC/MS | mg/L | 0.1 | <0.1 | <0.1 |
| Butyric Acid | | US EPA 624 mod | GC/MS | mg/L | 0.1 | <0.1 | <0.1 |
| Valeric Acid | | US EPA 624 mod | GC/MS | mg/L | 0.1 | <0.1 | <0.1 |
| Hexanoic Acid | | US EPA 624 mod | GC/MS | mg/L | 0.1 | <0.1 | <0.1 |
| Phenol | | MEWAM | HPLC | mg/L | 10 | <10 | <10 |
| m/p/o-Cresol | | MEWAM | HPLC | mg/L | 10 | <10 | <10 |
| Dimetheylphenols | | MEWAM | HPLC | mg/L | 10 | <10 | <10 |
| 2,3,5-Trimethylphenol | | MEWAM | HPLC | mg/L | 10 | <10 | <10 |
| 1-Naphthol | | MEWAM | HPLC | mg/L | 10 | <10 | <10 |
| 2-Isopropylphenol | | MEWAM | HPLC | mg/L | 10 | <10 | <10 |
| Hydroquinone | | MEWAM | HPLC | mg/L | 10 | <10 | <10 |
| Resorcinol | | MEWAM | HPLC | mg/L | 10 | <10 | <10 |
| H_2_S | | In-house | Indicator paper | N/A | N/A | Not Detected | Not Detected |
