# Supplementary figures and images for: Syntrophic Hydrocarbon Degradation in a Decommissioned Off-Shore Subsea Oil Storage Structure
Source: Microorganisms. 2021 Feb 11;9(2):356. doi: 10.3390/microorganisms9020356 (PMC7916938; doi:10.3390/microorganisms9020356)

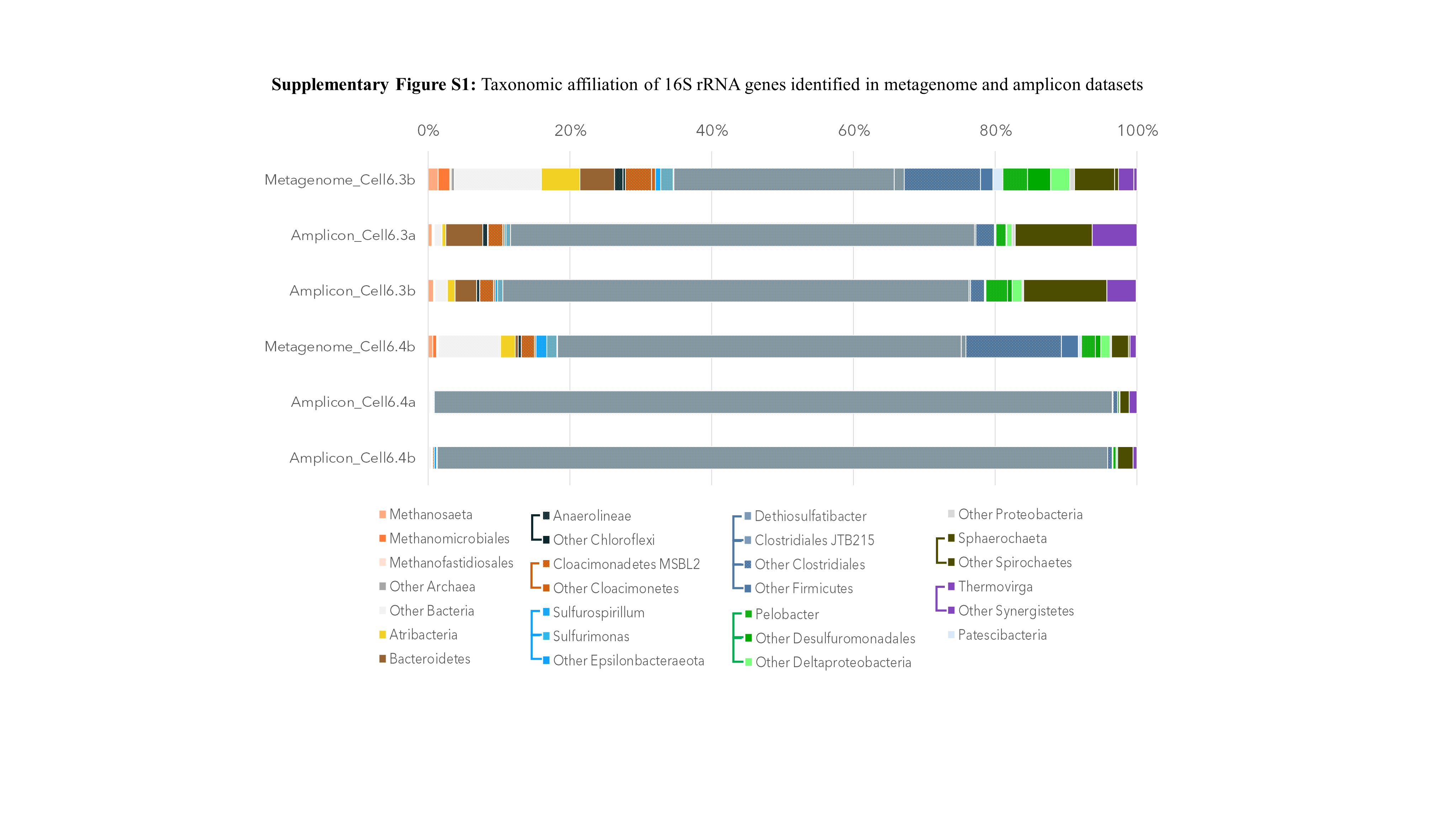

Supplement: Supplementary file 1 [file microorganisms-09-00356-s001.zip › Supplementary Figure S1.tif]
